# Superficial and multiple calcifications and ulceration associate with intraplaque hemorrhage in the carotid atherosclerotic plaque

**DOI:** 10.1007/s00330-018-5535-7

**Published:** 2018-06-06

**Authors:** Jia Yang, Xiangjun Pan, Bai Zhang, Yanhong Yan, Yabo Huang, Adam K. Woolf, Jonathan H Gillard, Zhongzhao Teng, Pinjing Hui

**Affiliations:** 1 Department of Stroke Center, The First Affiliated Hospital of Soochow University, 188 Shizi Street, Suzhou, 215006 China; 2 Department of Medicine, University of Cambridge, Cambridge, UK; 3 Department of Radiology, University of Cambridge, Level 5, Box 218, Addenbrooke’s Hospital, Hills Rd., Cambridge, CB2 0QQ UK

**Keywords:** Stroke, Artery, Atherosclerosis, Calcium

## Abstract

**Objective:**

Intraplaque hemorrhage (IPH) and ulceration of carotid atherosclerotic plaques have been associated with vulnerability while calcification has been conventionally thought protective. However, studies suggested calcification size and location may increase plaque vulnerability. This study explored the association between calcium configurations and ulceration with IPH.

**Methods:**

One hundred thirty-seven consecutive symptomatic patients scheduled for carotid endarterectomy were recruited. CTA and CTP were performed prior to surgery. Plaque samples were collected for histology. According to the location, calcifications were categorized into superficial, deep and mixed types; according to the size and number, calcifications were classified as thick and thin, multiple and single.

**Results:**

Seventy-one plaques had IPH (51.8%) and 83 had ulceration (60.6%). The appearance of IPH and ulceration was correlated (*r* = 0.49; *p* < 0.001). The incidence of multiple, superficial and thin calcifications was significantly higher in lesions with IPH and ulceration compared with those without. After adjusting factors including age, stenosis and ulceration, the presence of calcification [OR (95% CI), 3.0 (1.1-8.2), *p* = 0.035], multiple calcification [3.9 (1.4-10.9), *p* = 0.009] and superficial calcification [3.4 (1.1-10.8), *p* = 0.001] were all associated with IPH. ROC analysis showed that the AUC of superficial and multiple calcifications in detecting IPH was 0.63 and 0.66, respectively (*p* < 0.05). When the ulceration was combined, AUC increased significantly to 0.82 and 0.83, respectively. Results also showed that patients with lesions of both ulceration and IPH have significantly reduced brain perfusion in the area ipsilateral to the infarction.

**Conclusions:**

Superficial and multiple calcifications and ulceration were associated with carotid IPH, and they may be a surrogate for higher risk lesions.

**Key Points:**

*• CTA-defined superficial and multiple calcifications in carotid atherosclerotic plaques are independently associated with the presence of intraplaque hemorrhage.*

*• The combination of superficial and multiple calcifications and ulceration is highly predictive of carotid intraplaque hemorrhage.*

*• Patients with lesions of both ulceration and intraplaque hemorrhage have significantly reduced brain perfusion in the area ipsilateral to the infarction.*

## Introduction

Stroke has become the leading cause of death and adult disability in China [1, 2]. An atherosclerotic lesion located in the carotid circulation is responsible for 20~30% of ischemic stroke, mostly due to plaque rupture [3, 4]. It has been demonstrated that intraplaque hemorrhage (IPH) is associated with plaque progression and instability [5, 6]. The involvement of hemoglobin-rich plaque hemorrhage in the transformation from stable to unstable lesions was proposed over 80 years ago [7], the rupture of fragile neovessels being thought to initiate and promote IPH development [8]. IPH induces intraplaque inflammation by recruiting inflammatory contents [9], e.g., macrophages, while chronic inflammation links to calcium deposits [10]. As a result, IPH has been considered a biomarker for plaque instability [9]. Plaque ulceration is another high-risk feature; though much more common in symptomatic than asymptomatic individuals, it commonly leads to fibrous cap rupture and therefore carries an increased risk of ischemic cerebrovascular events [11, 12]. Combined, the presence of both IPH and ulceration carries the highest risk for recurrent ischemic events [13, 14]. Studies in the past ~20 years showed that high-resolution magnetic resonance imaging (hrMRI) can accurately identify these two features [6, 15].

Conversely, calcification has traditionally been thought protective. A systemic review of 24 studies found that asymptomatic patients have more calcium deposits in carotid plaques than symptomatic ones [16], supporting earlier observations [17, 18] that calcification was a sign of plaque stability. However, more recently studies have highlighted a controversial role of calcification with certain sizes [19, 20] and locations [21] being associated with increased plaque vulnerability.

Compared with hrMRI, computed tomography angiology (CTA) has less capacity to differentiate soft plaque components, e.g., IPH, while being particularly good at detecting calcium. Moreover, CTA is a more widely available modality than hrMRI. It would therefore be clinically advantageous to extend the use of CTA beyond its current remit, assessing luminal stenosis, and, by assessment of the calcium burden, predict a plaque’s vulnerability. Detailed analysis of the relationship between calcium and factors associated with plaque stability are therefore required to better understand carotid atherosclerotic risk. This study is designed as a comprehensive evaluation of the relationship between different features of calcification and ulceration with IPH.

## Materials and Methods

### Study population

This study was approved by the local ethics committee and all enrolled subjects signed written informed consent. The inclusion criteria [22] were symptomatic patients with luminal stenosis ≥ 70% defined by ultrasonography [North American Symptomatic Carotid Endarterectomy Trial (NASCET) criterion] [23] and luminal stenosis < 70% but with recurrent infarcts in the ipsilateral hemisphere despite optimal medical therapy. Symptomatic lesions were defined as ipsilateral ischemic events, including cerebral infarction, transient ischemic attack (TIA) and amaurosis fugax within 6 months. Exclusion criteria were patients with conditions that might increase high-sensitivity C-reactive protein (hs-CRP) levels [24], including severe peripheral arterial disease and apparent infection, and those with acute coronary syndromes who had undergone surgery within 90 days. A total of 137 consecutive patients scheduled for carotid endarterectomy (CEA) in the First Affiliated Hospital of Soochow University were recruited from February 2013 to August 2017. Patient demographics are listed in Table 1.

**Table 1:** Patient demography

Subject characteristic	Patients (*n* = 137)
Age (mean) (SD) (year)	68.8 (7.3)
Male (*n*) (%)	121 (88.3)
Hypertension (*n*) (%)	104 (75.9)
Hyperlipidemia (*n*) (%)	92 (67.2)
Diabetes (*n*) (%)	48 (35.0)
BMI (≥ 25) (*n*) (%)	63 (46.0)
Current or prior smoker (*n*) (%)	51 (37.2)
Cerebral infarction or TIA (*n*) (%)	81 (59.1)
Coronary artery disease (*n*) (%)	28 (20.4)
hs-CRP (median) [interquartile range] (mg/l)	2.5 [1.1-7.2]
Scr (median) [IQR] (μmol/l)	72.0 [63.0-85.5]
Statin use (*n*) (%)	74 (54.0)
Antiplatelet use (*n*) (%)	73 (53.3)

Hyperlipidemia was defined as serum low-density lipoprotein (LDL) cholesterol > 3.36 mmol/l or total cholesterol (TC) > 5.72 mmol/l or triglycerides (TG) > 2.3mmol/l

BMI, body mass index; TIA, transient ischemic attack; hs-CRP, high-sensitivity C-reactive protein; Scr, serum creatinine

### CTA and CTP imaging

Computerized tomography (CT) scanning was performed using a second-generation, dual-source 64-slice CT scanner (Somatom Definition Flash, Siemens Healthcare, Forchheim, Germany). The cross-sectional helical scan was performed followed by the CT perfusion (CTP) scan with a 10-cm range covering the basal ganglia and adjacent layers. The tube voltage was 80 kV with a current of 150 mAs. Contrast agent (iohexol, 350mg I/ml) 40 ml and saline 60 ml were injected via a cubital vein at a rate of 6.0 ml/s using a high-pressure syringe (Germany Ulrich Automatic contrast agent syringe). The scan started in 8 s after the injection, and the scanning duration was 37 s. About 400 frames were acquired from each patient.

The dual-source CTA scan of the head and neck from the aortic arch to the top of the skull with a slice thickness of 0.5 mm was performed 5 min after the CTP scan. A total of 60 ml contrast agent (iohexol, 350 mg I/ml) and the subsequent 40 ml saline solution were injected at a flow rate of 5 ml/s into the same cubital vein with the high-pressure syringe system. The bolus-tracking technique was used for image acquisition with a region of interest in the common carotid artery and a trigger at 100 HU. Data acquisition was initiated after the threshold was reached in the common carotid artery. The carotid CTA scanning parameters were: rotation speed of 0.33 s/rev, alignment of 64 × 0.6 mm^2^, voltages of 140 kV and 80 kV, respectively, current of 56 mAs and 234 mAs, pitch of 0.65, field of view of 230 mm and reconstruction thickness of 1 mm. All CTA images were sent to a post-processing workstation (Syngo.via) and reconstructed with a thickness of 1 mm.

### CTA and CTP imaging review

All images were reviewed and analyzed using the Neuro VPCT package (Siemens Healthcare, Forchheim, Germany). Cerebral blood flow (CBF), cerebral blood volume (CBV), mean transit time (MTT) and time to peak (TTP) were extracted for analysis. Multiplanar reformation, curved planar reformation, maximum intensity projection and volume rendering were used for the characterization of carotid plaque.

**Fig. 1:**
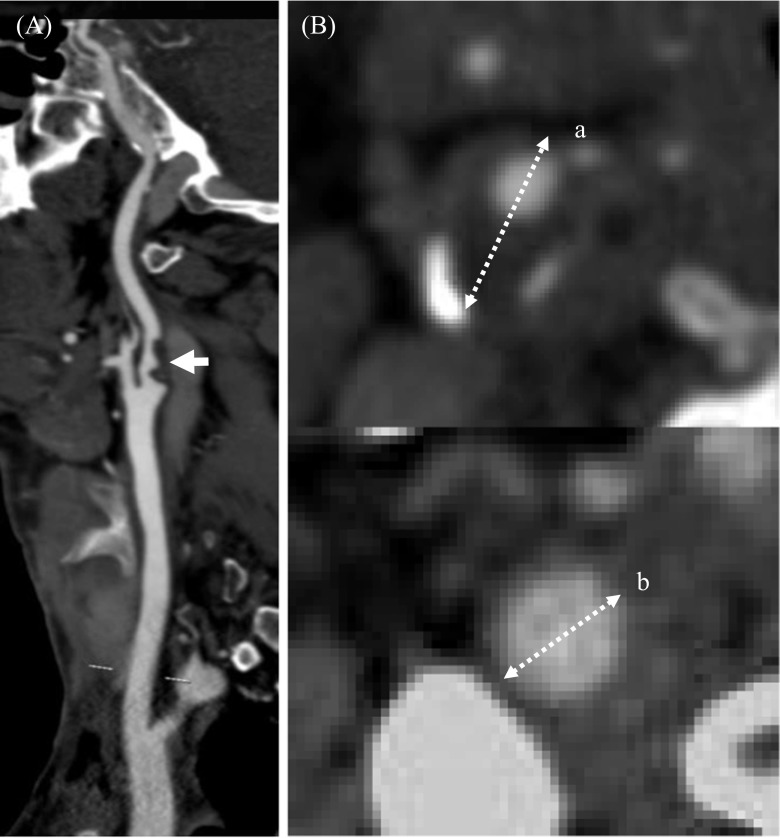
Plaque ulceration and remodeling. A Presence of ulceration (arrow) was identified. B Remodeling index (RI) was computed as a/b

**Fig. 2:**
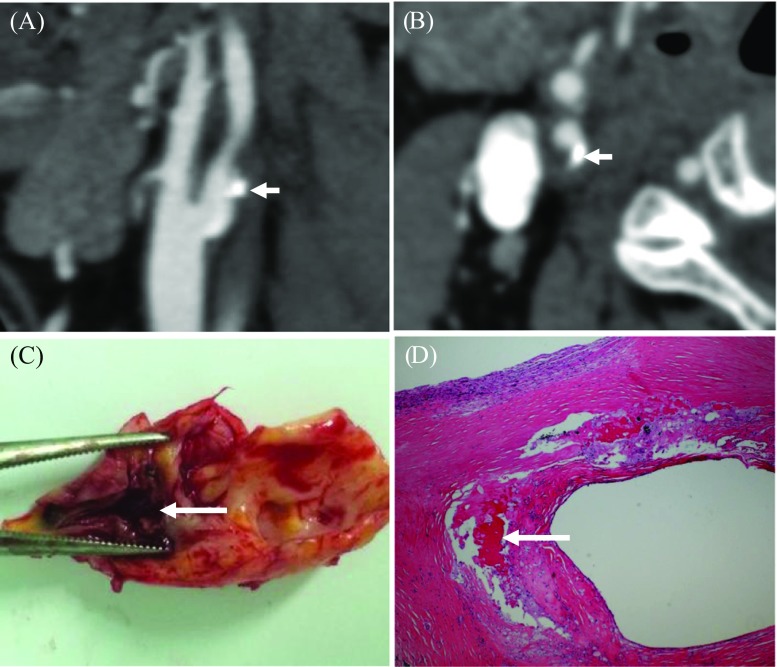
CTA and pathological image showing superficial calcification and IPH on the internal carotid artery. **A** and **B** MPR images of the plaque with a calcified nodule located at the intimal-lumen interface (arrow; **B** is an axial view of **A** at the level with calcification). **C** Eyeball assessment of the CEA specimen with IPH. **D** Hematoxylin and eosin (×16) stain showing IPH (arrow)

**Fig. 3:**
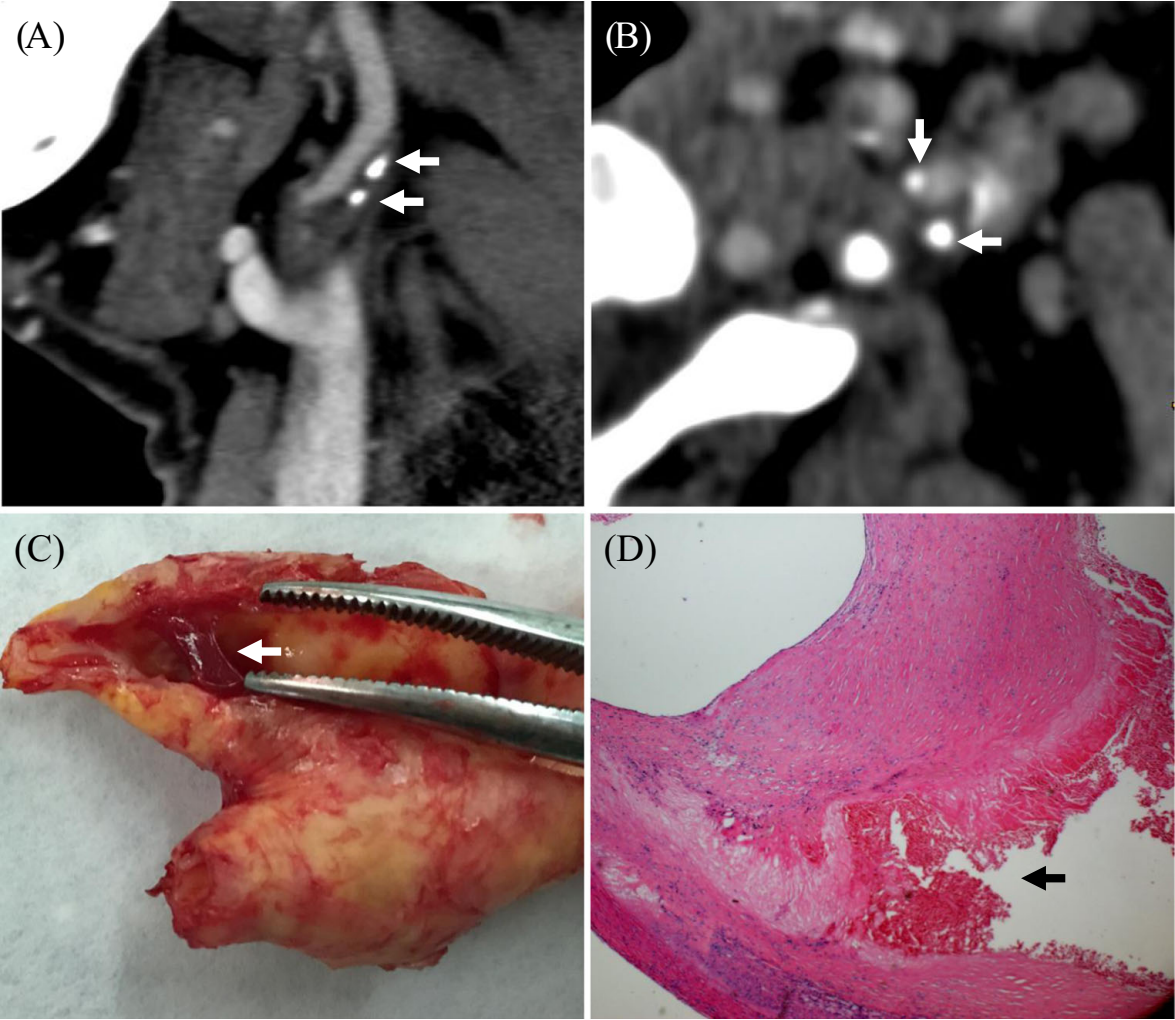
CTA and pathological image showing multiple calcifications and IPH on the internal carotid artery. **A** and **B** MPR images of the plaque with multiple calcified nodules within the plaque (arrows; **B** is an axial view of **A** at the level with calcifications). **C** Eyeball assessment of the CEA specimen showing IPH (arrow). **D** Hematoxylin and eosin (×16) stain showing IPH (arrow)

The degree of luminal stenosis, plaque thickness, surface ulceration and remodeling index (RI = the maximum wall thickness at the most stenotic site/the wall thickness at the nearby disease-free site) were calculated or identified. Positive soft plaque was defined as maximum soft plaque thickness >2 mm [25], and a positive remodeling index was reported as RI ≥ 1.1 [26]. The mean plaque Hounsfield unit (HU) was defined as the average HU in the region between the lumen and outer wall contour across the whole plaque volume [27]. Ulceration was defined as irregular concavities with a minimum depth of 1 mm on any plane [28, 29] (Fig. 1). The presence of calcification and the location and number of calcific nodules were recorded and categorized as either surface (Fig. 2), deep or mixed calcification. Superficial calcification was defined as a calcified nodule located at the intimal-lumen interface or close to the lumen [30]. Deep calcification was defined as a calcified nodule located at the media/adventitia border or close to the adventitia. The presence of both deep and superficial calcification was regarded as a mixed category. According to the maximum thickness, calcification was classified as either thick (≥ 2 mm) or thin (< 2 mm). A distinction was also made between single and multiple nodules (Fig. 3). All parameters and characteristics were independently evaluated by two experienced radiologists, both of whom were blinded to patient clinical presentation and histopathology findings.

### Histopathologic evaluation of plaque samples

Plaque samples were cut into proximal and distal parts at the most stenotic site and immediately fixed in 4% buffered formalin for 48 h, then decalcified and embedded in paraffin. Further transverse sections of 4 μm thickness at a 1-mm interval were obtained. Hematoxylin and eosin staining was used to identify IPH according to previously published criteria [31, 32]. Sections were then assessed by two independent histopathologists blinded to the clinical and imaging findings. The presence or absence of IPH was recorded.

### Statistical analysis

Statistical analysis was performed with SPSS 19.0 (IBM Corp., Armonk, NY, USA). Continuous quantitative variables were described as mean ± SD or median [interquartile range, IQR] depending on the distribution, while categorical variables were described as percentages. The Student’s *t*, Mann-Whitney U, χ2 and Fisher’s exact tests were used to compare baseline characteristics, CTP parameters and CTA makers between patients with and without IPH/ulceration where appropriate. Multivariate regression analysis was used to explore associations between characteristic calcifications and IPH and ulceration. To assess the discriminatory potential of each marker or combination of markers in identifying IPH, the area under the curve (AUC) of the receiver-operating characteristic (ROC) were reported with 95% confidence intervals (CI). The interobserver agreement was calculated using an intraclass correlation coefficient or Cohen’s κ statistics based on 20 randomly chosen patients assessed by observer A and observer B. A *p* value < 0.05 was assumed to be statistically significant.

## Results

### Patient characteristics

One hundred thirty-seven patients were recruited with a mean age of 68.8 ± 7.3 years; 121 (88.3%) were male; 104 (75.9%) had hypertension, 48 (35.0%) diabetes and 92 (78.8%) hyperlipidemia. The majority were receiving medications prior to the study (54.0% on statin, 53.3% on antiplatelet). More patient demographics are shown in Table 1.

### Clinical risk factors, IPH, ulceration and brain perfusion

IPH was found in 71 (51.8%) patients and ulceration in 83 (60.6%). Sixty-two plaques (45.2%) had both IPH and ulceration, and 45 (32.8%) had neither of these features. The appearance of IPH and ulceration was significantly correlated (*r* = 0.49; *p* < 0.001).

As opposed to patients without carotid IPH and ulceration, those with tended to be both older (70.9 ± 6.7 versus 66.5 ± 7.3 years, *p* < 0.001; 70.3 ± 7.2 versus 66.5 ± 6.8 years, *p* = 0.003, respectively) and have a significantly higher level of serum hs-CRP (3.6 mg/l versus 1.7 mg/l, *p* = 0.001; 4.9 mg/l versus 1.2 mg/l, *p* < 0.001). Moreover, hypertension was more likely to be present in patients with carotid plaque surface ulceration than those without (88.0% versus 57.4%, *p* < 0.001). Other clinical risk factors did not differ significantly between these two groups as shown in Table 2.

**Table 2:** Clinical characteristics between groups with and without IPH and ulceration

Clinical characteristics	Ulceration (+) (*n* = 83)	Ulceration (-) (*n* = 54)	*p* value	IPH (+) (*n* = 71)	IPH (-) (*n* = 71)	*p* value
Age (mean) (SD) (year)	70.3 (7.2)	66.5 (6.8)	0.003	70.9 (6.7)	66.5 (7.3)	< 0.001
Male (*n*) (%)	76 (91.6)	45 (83.3)	0.143	62 (87.3)	59 (89.4)	0.706
Hypertension (*n*) (%)	73 (88.0)	31 (57.3)	< 0.001	57 (80.3)	47 (71.2)	0.215
Hyperlipidemia (*n*) (%)	55 (66.3)	37 (68.5)	0.784	50 (70.4)	42 (63.6)	0.398
Diabetes (*n*) (%)	33 (39.8)	15 (27.8)	0.151	26 (36.6)	22 (33.3)	0.687
BMI (≥ 25) (*n*) (%)	43 (51.8)	20 (37.0)	0.090	37 (52.1)	26 (39.4)	0.136
Current or prior smoker (*n*) (%)	35 (42.2)	16 (29.6)	0.138	29 (40.8)	22 (33.3)	0.363
Cerebral infarction or TIA (*n*) (%)	53 (63.9)	28 (51.9)	0.163	45 (63.4)	36 (54.4)	0.293
Coronary artery disease (*n*) (%)	18 (21.7)	10 (18.5)	0.653	15 (21.1)	13 (19.7)	0.836
hs-CRP (median) [IQR] (mg/l)	4.9 [1.8-11.4]	1.2 [0.6-2.2]	< 0.001	3.6 [1.6-11.1]	1.7 [0.8-5.0]	0.001
Scr (median) [IQR] (μcr /l)	73.0 [62.0-85.0]	72.0 [65.0-88.0]	0.925	72.0 [63.0-85.0]	74.0 [63.0-88.0]	0.649
Statin use (*n*) (%)	41 (49.4)	33 (61.1)	0.179	36 (50.7)	38 (57.6)	0.420
Antiplatelet (*n*) (%)	47 (56.6)	26 (48.1)	0.331	43 (60.6)	30 (45.5)	0.077

BMI, body mass index; TIA, transient ischemic attack; hs-CRP, high-sensitivity C-reactive protein; Scr, serum creatinine

IPH/ulceration (+) or IPH/ulceration (-), carotid plaques with or without intraplaque hemorrhage/ulceration

**Fig. 4:**
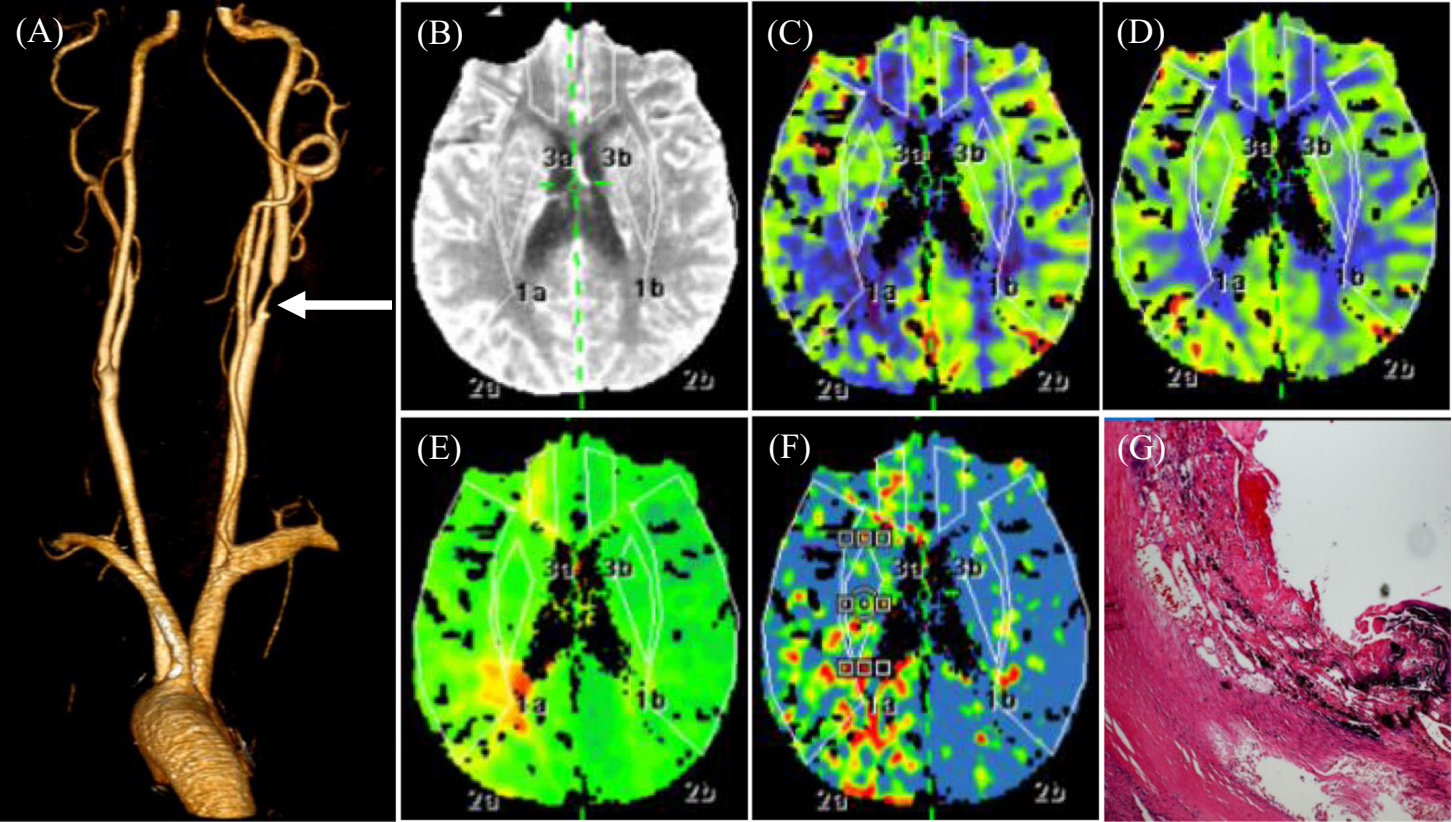
CTA, CTP and the pathological image showing brain perfusion and ipsilateral IPH. **A** A volume-rendering image shows severe stenosis at the beginning of the right internal carotid artery (arrow). **B**-**F** CBF, CBV significantly decreased; TTP and MTT prolonged on the right temporal lobe, suggesting right temporal lobe ischemia. **G** Hematoxylin and eosin stain (×16) showing IPH and ruptured fibrous cap

CTP parameters between groups with and without IPH were compared, as listed in Table 3 with an example shown in Fig. 4. Median CBF between groups with and without IPH was significantly different (54.7 [48.2-60.6] versus 56.5 [51.7-66.4], *p* = 0.028). However, no significant difference was found in CBV, TTP and MTT. Median TTP and MTT for the group with ulceration were seen to be longer than in the group without (11.2 [9.8-12.8] versus 10.1 [9.4-12.4], *p* = 0.041; 4.0 [3.7-4.7] vs. 3.8 [3.5-4.4], *p* = 0.045). On the contrary, CBF and CBV showed no significant difference between the groups with and without ulceration (Table 3).

**Table 3:** CTP parameters between groups with and without IPH and ulceration (median [IQR])

CTP parameters	Ulceration (+) (*n* = 83)	Ulceration (-) (*n* = 54)	*p* value	IPH (+) (*n* = 71)	IPH (-) (*n* = 66)	*p* value
CBF (ml/100 ml/min)	55.2 [48.9-61.6]	57.0 [51.1-65.6]	0.166	54.7 [48.2-60.6]	56.5 [51.7-66.4]	0.028
CBV (ml/100 ml)	3.3 [2.9-3.5]	3.4 [3.0-3.7]	0.186	3.3 [2.9-3.5]	3.4 [3.0-3.7]	0.089
TTP (s)	11.2 [985-12.8]	10.1 [9.4-12.4]	0.041	10.5 [9.5-12.8]	10.8 [9.6-12.6]	0.826
MTT (s)	4.0 [3.7-4.7]	3.8 [3.5-4.4]	0.045	4.0 [3.6-4.7]	3.8 [3.5-4.3]	0.188

CBF, cerebral blood flow; CBV, cerebral blood volume; TTP, time to peak; MTT, mean transit time; Ulceration/IPH (+), carotid plaques with ulceration or intraplaque hemorrhage; Ulceration/IPH (-), carotid plaques without ulceration or intraplaque hemorrhage

### The association of CTA-identified plaque features, ulceration and IPH

As shown in Table 4, lesions with IPH showed a higher prevalence of plaque ulceration than those without (87.3% versus 31.8%, *p* < 0.001). The prevalence of calcification, multiple calcifications, superficial calcification, mixed calcifications and thin calcification was significantly higher in plaques with IPH compared with those without (83.1% versus 60.6%, *p* = 0.003; 52.1% versus 19.7%, *p* < 0.001; 36.6% versus 10.6%, *p* < 0.001; 22.5% versus 9.1%, *p* = 0.044; 45.1% versus 24.2%, *p* = 0.011; respectively). However, deep calcification was less prevalent in plaques with IPH compared with those without (23.9% versus 40.9%, *p* = 0.034). Moreover, mean plaque density, maximum total plaque thickness, the incidence of positive soft plaque, degree of stenosis and thick calcification showed no significant difference between the two groups.

**Table 4:** Carotid plaque CTA markers between groups with and without IPH and ulceration

CTA markers	Ulceration (+) (*n* = 83)	Ulceration (-) (*n* = 54)	*p* value	IPH (+) (*n* = 71)	IPH (-) (*n* = 66)	*p* value
NASCET-defined stenosis (mean) (SD) (%)	77.3 (11.5)	81.9 (10.3)	0.018	77.4 (11.9)	80.6 (10.4)	0.124
Ulceration (*n*) (%)	-	-	-	62 (87.3)	21 (31.8)	< 0.001
Positive remodeling (*n*) (%)	51 (61.4)	29 (53.7)	0.369	46 (64.8)	34 (51.5)	0.115
Mean plaque density (median) [IQR] (HU)	52 [41-66]	58 [45-66]	0.342	52 [43-65]	57 [44-66]	0.290
Maximum total plaque thickness (mean) (SD) (mm)	4.2 (0.9)	4.1 (1.0)	0.494	4.0 (0.8)	4.3 (1.0)	0.131
Positive soft plaque (*n*) (%)	57 (68.7)	35 (64.8)	0.638	52 (73.2)	40 (60.6)	0.116
Presence of calcification (*n*) (%)	64 (77.1)	35 (64.8)	0.116	59 (83.1)	40 (60.6)	0.003
Multiple calcification (*n*) (%)	39 (47.0)	11 (20.4)	0.002	37 (52.1)	13 (19.7)	< 0.001
Single calcification (*n*) (%)	22 (26.5)	23 (42.6)	0.050	18 (28.2)	27 (44.3)	0.053
Superficial calcification (*n*) (%)	27 (32.5)	6 (11.1)	0.004	26 (36.6)	7 (10.6)	< 0.001
Deep calcification (*n*) (%)	20 (24.1)	24 (44.4)	0.013	17 (23.9)	27 (40.9)	0.034
Mixed calcification (*n*) (%)	17 (20.5)	5 (9.3)	0.080	16 (22.5)	6 (9.1)	0.032
Thick calcification (*n*) (%)	27 (32.5)	24 (44.4)	0.159	27 (38.0)	24 (36.4)	0.840
Thin calcification (*n*) (%)	37 (44.6)	11(20.4)	0.004	32 (45.1)	16 (24.2)	0.011

Positive soft plaque indicates maximum soft plaque thickness > 2 mm; positive remodeling, remodeling index > 1.1

IPH/ulceration (+) or IPH/ulceration (-), carotid plaques with or without intraplaque hemorrhage/ulceration

Compared with plaques without ulceration, those with ulceration showed less severe luminal stenosis (NASCET-defined stenosis, 77.3% versus 81.9%, *p* = 0.018), and the incidence of calcification, multiple calcifications, superficial calcification and thin calcification was significantly higher, while deep calcification was significantly less likely in carotid plaques with ulceration (all *p* < 0.05; Table 4).

In the univariate regression analysis (Table 5), the presence of calcification, multiple calcifications, superficial calcification, deep calcification, mixed calcifications and thin calcification was all significantly associated with IPH, but not single and thick calcifications. After adjusting for age, NASCET-defined stenosis, mean plaque HU, plaque maximum thickness, positive remodeling, positive soft plaque and ulceration, the associations remained for the presence of calcification, multiple calcification and superficial calcifications (Table 5).

**Table 5:** Relationship between calcification characteristics and IPH

Calcification characteristics	Univariate	*p* value	Multivariate	*p* value
	OR (95% CI)		OR (95% CI)	
Presence of calcification	3.2 (1.4-7.1)	0.004	3.0 (1.1-8.2)	0.035
Multiple calcifications	4.3 (2.0-9.3)	< 0.001	3.9 (1.4-10.9)	0.009
Single calcification	0.5 (0.2-1.0)	0.050	0.6 (0.2-1.4)	0.217
Superficial calcification	4.9 (1.9-12.2)	0.001	3.4 (1.1-10.8)	0.035
Deep calcification	0.5 (0.2-0.9)	0.035	0.6 (0.2-1.7)	0.377
Mixed calcification	2.9 (1.1-8.0)	0.038	2.0 (0.6-7.1)	0.262
Thick calcification	1.1 (0.5-2.1)	0.840	2.3 (0.8-6.2)	0.106
Thin calcification	2.6 (1.2-5.3)	0.012	1.2 (0.5-3.1)	0.710

Multivariate: Adjusted for age, NASCET-defined stenosis, mean plaque HU, plaque maximum thickness, positive remodeling, positive soft plaque and ulceration

OR, odds ratio; CI, confidence interval

Receiver-operating characteristic (ROC) analysis showed the different features’ capacity to identify IPH. The AUC for superficial calcification was 0.63 (0.54-0.72) (95% confidence interval), while for multiple calcifications was 0.66 (0.57-0.75) and for ulceration 0.78 (0.70-0.86). When ulceration was combined with either superficial calcification or multiple calcifications, the AUC increased significantly [0.82 (0.74-0.88); 0.83 (0.76-0.89), respectively; both *p* < 0.001].

### Interobserver agreement

Data from 20 randomly selected patients was used to assess the interobserver agreement. The interobserver intraclass correlation coefficient for mean plaque HU was 0.92 (0.80-0.97) and 0.89 (0.75-0.96) for plaque maximum thickness. The Cohen κ between two reviewers for presence of ulceration was 0.90 (0.80-0.99), 0.89 (0.78-0.99) for positive soft plaque and 0.76 (0.60-0.92) for calcification. The interobserver agreement of CTP was excellent for CBF [κ: 0.85 (0.66-0.94)], TTP [κ: 0.95 (0.89-0.98)] and MTT [κ: 0.91 (0.79-0.96)], but moderate for CBV [κ: 0.61 (0.23-0.83)].

## Discussion

Carotid plaque IPH has been considered an emerging biomarker to predict future ischemic cerebrovascular events [33–35]. hrMRI has an excellent capacity to demonstrate IPH, and many T1-weighted clinical sequences including 3D-TOF and MPRAG can detect the finding [36, 37], while CTA is less useful for differentiating soft contents, such as IPH, lipid and fibrous tissues. However, CTA is more commonly available in clinical practice, being significantly faster, relative to hrMRI, with different and fewer contraindications than MR imaging in general. CTA imaging has favorable properties of its own, in particular its ability to define and characterize calcification [38, 39] though still a relatively controversial marker of plaque instability. Because of its ease and availability, the use of CTA for the purpose of categorizing high-risk plaque is appealing. As shown in this study, calcium burden and ulceration, readily identifiable on CTA, are associated with higher risk plaque features such as IPH. The results obtained show CTA defined superficial and multiple calcification in particular to be associated with IPH both before and after adjusting confounding factors. Interestingly, the association was not seen with thick calcification. The results imply CTA-defined superficial and multiple calcifications may be useful indirect biomarkers for the identification of higher risk lesions, those containing IPH, before hrMRI becomes more readily available. Although for the detection of IPH, specific carotid surface coils and MR sequences are not mandatory, because of the small size of IPH in the lesion, dedicated multi-channel carotid surface coil and MR sequences, e.g., a direct thrombus imaging sequence, MPRAGE (magnetization-prepared rapid acquisition gradient-echo) and SNAP (simultaneous non-contrast angiography and intraplaque hemorrhage), are recommended.

Although in general calcium is considered a protective factor, increasing evidence suggests size [20, 40] and location may influence its effect and in certain configurations increase vulnerability. In a study of 63 patients by Xu et al. [41], certain locations of hrMRI-defined calcium were found to be associated with IPH plaque. This was confirmed in a study using time of flight and MPRAGE where IPH was more likely to be found in lesions with superficial compared with deep calcium [42], despite the fact that the extremely short T1 value of MRI makes calcium detection challenging. Although ultrashort echo time (UTE) [43] and gray blood [44] MR sequences have been developed for the detection of calcium, the availability of such imaging techniques is limited.

The underlying mechanism behind the relationship of IPH and superficial calcification in carotid plaques is unclear. It may be that calcium, being much stiffer than the soft components such as the fibrous cap, introduces a stiffness mismatch within the structure when present. This mismatch will create a high stress concentration when calcium is located in the region closest to the lumen or directly on the lumen surface [21, 45]. High mechanical loading then has the potential to damage local tissues, particularly the thinner fibrous caps and fragile walls of neovessels, thereby promoting the formation and development of IPH and plaque rupture [46]. On the other hand, deep calcification being further away from the lumen has little or no impact on plaque stress. Moreover, it could act as a barrier to the growth of the vasa vasorum from the adventitia to the intima preventing IPH formation. Our study went on to show the association of multiple calcifications with carotid IPH, which may be attributable to a similar mechanism. In case of multiple calcium nodules, in particular where two nodules are close together, connective tissues between them will be subject to high mechanical loading. As a consequence, the local disruption and potential for rupture may exist.

In this study, a weak correlation was found between the size (i.e., the maximum calcification thickness) of calcification and IPH within the carotid plaque. This is different from findings of previous studies where the volume and length of calcifications were independently associated with IPH and major adverse cardiovascular events [20, 40]. Results obtained from this study showed that age and hs-CRP level were associated with both IPH and ulceration. This was in agreement with previous findings that IPH was found more often in elderly patients and those with increased hs-CRP levels [24, 47].

The strong association of ulceration and IPH demonstrated in this study agreed with previous findings [27]. Both IPH and ulceration were found in 62 patients (45.3%), only 9 plaques (6.6%) had IPH but no ulceration, 21 plaques (15.3%) showed ulceration but no IPH, and neither IPH nor ulceration was found in 45 plaques (32.8%). Superficial calcification and multiple calcifications were observed more often in plaques with IPH and ulceration than those with neither IPH nor ulceration.

This study shows how detailed plaque characteristics may associate with brain perfusion (Table 3). It was observed that patients with lesions containing IPH have lower CBF compared with those without. For patients with ulcerated carotid plaque, there was a longer TTP and MMT in the ipsilateral infarction area. Supporting this, the study found that patients with lesions with both IPH and ulceration had poorer brain perfusion in the ipsilateral infarction area compared with those containing one or neither of them. However, it must be pointed out that the measurement of the perfusion parameter might be observer and patient condition dependent. Further studies are therefore needed to validate the association identified in this study.

Despite the interesting findings of this study, limitations exist. This is a cross-sectional retrospective study, and the cause-effect of IPH and calcium features cannot be determined. Second, only symptomatic patients scheduled for CEA were recruited in this study, and it is therefore unclear whether either asymptomatic patients or those not scheduled for CEA would show the same associations. Third, the study only considered the presence/absence of IPH, not the IPH volume, location and age. Finally, although CTA remains an ideal imaging examination for the detection of calcification, due to the bloom effect seen with CTA, typing of calcium can occasionally be challenging.

## Abbreviations

CBF: Cerebral blood flow
CBV: Cerebral blood volume
CEA: Carotid endarterectomy
CT: Computed tomography
CTA: CT angiology
CTP: CT perfusion
hrMRI: High-resolution magnetic resonance imaging
hs-CRP: High-sensitivity C-reactive protein
IPH: Intraplaque hemorrhage
IQR: Interquartile range
MTT: Mean transit time
NASCET: North American Symptomatic Carotid Endarterectomy Trial
OR: Odds ratio
RI: Remodeling index
TIA: Transient ischemic attack
TTP: Time to peak
UTE: Ultrashort echo time

## References

[1] (2015) Global, regional, and national age-sex specific all-cause and cause-specific mortality for 240 causes of death, 1990-2013: a systematic analysis for the Global Burden of Disease Study 2013. Lancet 385:117-171 10.1016/S0140-6736(14)61682-2 PMC4340604 25530442

[2] Liu Liping, Wang David, Wong K.S. Lawrence, Wang Yongjun (2011). Stroke and Stroke Care in China. Stroke.

[3] Redgrave JN, Lovett JK, Gallagher PJ, Rothwell PM (2006). Histological assessment of 526 symptomatic carotid plaques in relation to the nature and timing of ischemic symptoms: the Oxford plaque study. Circulation.

[4] von SB, Lüdemann J, Völzke H, Dörr M, Kessler C, Schminke U (2010). Common carotid intima-media thickness and framingham risk score predict incident carotid atherosclerotic plaque formation: longitudinal results from the study of health in Pomerania. Stroke.

[5] Sun J, Song Y, Chen H (2013). Adventitial perfusion and intraplaque hemorrhage: a dynamic contrast-enhanced MRI study in the carotid artery. Stroke.

[6] Takaya N, Yuan C, Chu B (2005). Presence of intraplaque hemorrhage stimulates progression of carotid atherosclerotic plaques: a high-resolution magnetic resonance imaging study. Circulation.

[7] Paterson JC (1936). Vascularization and hemorrhage of the intima of coronary atherosclerotic arteries. Arch Pathol.

[8] Wartman WB (1938). Occlusion of the coronary arteries by hemorrhage into their walls. Am Heart J.

[9] Teng Z, Sadat U, Brown AJ, Gillard JH (2014). Plaque hemorrhage in carotid artery disease: pathogenesis. clinical and biomechanical considerations. J Biomech.

[10] Abedin M, Tintut Y, Demer LL (2004). Vascular calcification: mechanisms and clinical ramifications. Arterioscler Thromb Vasc Biol.

[11] Eliasziw M, Streifler JY, Fox AJ, Hachinski VC, Ferguson GG, Barnett HJ (1994). Significance of plaque ulceration in symptomatic patients with high-grade carotid stenosis. North American Symptomatic Carotid Endarterectomy Trial. Stroke.

[12] Fisher M, Paganini-Hill A, Martin A (2005). Carotid plaque pathology: thrombosis, ulceration. and stroke pathogenesis. Stroke.

[13] Kwee RM, van Oostenbrugge RJ, Mess WH (2013). MRI of carotid atherosclerosis to identify TIA and stroke patients who are at risk of a recurrence. J Magn Reson Imaging.

[14] Teng Z, Sadat U, Huang Y (2011). In vivo MRI-based 3D mechanical stress-strain profiles of carotid plaques with juxtaluminal plaque haemorrhage: an exploratory study for the mechanism of subsequent cerebrovascular events. Eur J Vasc Endovasc Surg.

[15] Underhill HR, Hatsukami TS, Fayad ZA, Fuster V, Yuan C (2010). MRI of carotid atherosclerosis: clinical implications and future directions. Nat Rev Cardiol.

[16] Kwee RM (2010). Systematic review on the association between calcification in carotid plaques and clinical ischemic symptoms. J Vasc Surg.

[17] Wahlgren CM, Zheng W, Shaalan W, Tang J, Bassiouny HS (2009). Human carotid plaque calcification and vulnerability. Relationship between degree of plaque calcification, fibrous cap inflammatory gene expression and symptomatology. Cerebrovasc Dis.

[18] Huang H, Virmani R, Younis H, Burke AP, Kamm RD, Lee RT (2001). The impact of calcification on the biomechanical stability of atherosclerotic plaques. Circulation.

[19] Vengrenyuk Y, Carlier S, Xanthos S (2006). A hypothesis for vulnerable plaque rupture due to stress-induced debonding around cellular microcalcifications in thin fibrous caps. Proc Natl Acad Sci U S A.

[20] van den Bouwhuijsen QJ, Bos D, Ikram MA (2015). Coexistence of Calcification, Intraplaque Hemorrhage and Lipid Core within the Asymptomatic Atherosclerotic Carotid Plaque: The Rotterdam Study. Cerebrovasc Dis.

[21] Teng Z, He J, Sadat U (2014). How does juxtaluminal calcium affect critical mechanical conditions in carotid atherosclerotic plaque?. An exploratory study. IEEE Trans Biomed Eng.

[22] Liapis CD, Bell PR, Mikhailidis D (2009). ESVS guidelines. Invasive treatment for carotid stenosis: indications, techniques. Eur J Vasc Endovasc Surg.

[23] (1991) North American Symptomatic Carotid Endarterectomy Trial. Methods, patient characteristics, and progress. Stroke 22:711-720 10.1161/01.str.22.6.711 2057968

[24] Albuquerque LC, Narvaes LB, Maciel AA (2007). Intraplaque hemorrhage assessed by high-resolution magnetic resonance imaging and C-reactive protein in carotid atherosclerosis. J Vasc Surg.

[25] Motoyama S, Sarai M, Harigaya H (2009). Computed tomographic angiography characteristics of atherosclerotic plaques subsequently resulting in acute coronary syndrome. J Am Coll Cardiol.

[26] Mosleh W, Adib K, Natdanai P (2017). High-risk carotid plaques identified by CT-angiogram can predict acute myocardial infarction. Int J Cardiovasc Imaging.

[27] U-King-Im JM, Fox AJ, Aviv RI (2010). Characterization of carotid plaque hemorrhage: a CT angiography and MR intraplaque hemorrhage study. Stroke.

[28] Saba L, Caddeo G, Sanfilippo R, Montisci R, Mallarini G (2007). CT and ultrasound in the study of ulcerated carotid plaque compared with surgical results: potentialities and advantages of multidetector row CT angiography. AJNR Am J Neuroradiol.

[29] Saba L, Caddeo G, Sanfilippo R, Montisci R, Mallarini G (2007). Efficacy and sensitivity of axial scans and different reconstruction methods in the study of the ulcerated carotid plaque using multidetector-row CT angiography: comparison with surgical results. AJNR Am J Neuroradiol.

[30] Mintz GS, Popma JJ, Pichard AD (1995). Patterns of calcification in coronary artery disease. A statistical analysis of intravascular ultrasound and coronary angiography in 1155 lesions. Circulation.

[31] Lusby RJ, Ferrell LD, Ehrenfeld WK, Stoney RJ, Wylie EJ (1982). Carotid plaque hemorrhage. Its role in production of cerebral ischemia. Arch Surg.

[32] Bassiouny HS, Davis H, Massawa N, Gewertz BL, Glagov S, Zarins CK (1989). Critical carotid stenoses: morphologic and chemical similarity between symptomatic and asymptomatic plaques. J Vasc Surg.

[33] Saam T, Hetterich H, Hoffmann V (2013). Meta-analysis and systematic review of the predictive value of carotid plaque hemorrhage on cerebrovascular events by magnetic resonance imaging. J Am Coll Cardiol.

[34] Chu B, Kampschulte A, Ferguson MS (2004). Hemorrhage in the atherosclerotic carotid plaque: a high-resolution MRI study. Stroke.

[35] Finn AV, Nakano M, Narula J, Kolodgie FD, Virmani R (2010). Concept of vulnerable/unstable plaque. Arterioscler Thromb Vasc Biol.

[36] Scott MJ, Yoon HC, Kim SE (2015). Carotid MRI detection of intraplaque hemorrhage at 3T and 1. 5T. J Neuroimaging.

[37] Ota H, Yarnykh VL, Ferguson MS (2010). Carotid intraplaque hemorrhage imaging at 3.0-T MR imaging: comparison of the diagnostic performance of three T1-weighted sequences. Radiology.

[38] Hong C, Becker CR, Schoepf UJ, Ohnesorge B, Bruening R, Reiser MF (2002). Coronary artery calcium: absolute quantification in nonenhanced and contrast-enhanced multi-detector row CT studies. Radiology.

[39] Wintermark M, Jawadi SS, Rapp JH (2008). High-resolution CT imaging of carotid artery atherosclerotic plaques. AJNR Am J Neuroradiol.

[40] Nonin S, Iwata S, Sugioka K (2017). Plaque surface irregularity and calcification length within carotid plaque predict secondary events in patients with coronary artery disease. Atherosclerosis.

[41] Xu X, Ju H, Cai J, Cai Y, Wang X, Wang Q (2010). High-resolution MR study of the relationship between superficial calcification and the stability of carotid atherosclerotic plaque. Int J Cardiovasc Imaging.

[42] Lin R, Chen S, Liu G, Xue Y, Zhao X (2017). Association between carotid atherosclerotic plaque calcification and intraplaque hemorrhage: a magnetic resonance imaging study. Arterioscler Thromb Vasc Biol.

[43] Chan CF, Keenan NG, Nielles-Vallespin S (2010). Ultra-short echo time cardiovascular magnetic resonance of atherosclerotic carotid plaque. J Cardiovasc Magn Reson.

[44] Koktzoglou I (2013). Gray blood magnetic resonance for carotid wall imaging and visualization of deep-seated and superficial vascular calcifications. Magn Reson Med.

[45] Li ZY, Howarth S, Tang T, Graves M, U-King-Im J, Gillard JH (2007). Does calcium deposition play a role in the stability of atheroma?. Location may be the key. Cerebrovasc Dis.

[46] Teng Z, He J, Degnan AJ (2012). Critical mechanical conditions around neovessels in carotid atherosclerotic plaque may promote intraplaque hemorrhage. Atherosclerosis.

[47] Derksen WJ, Peeters W, Tersteeg C (2011). Age and coumarin-type anticoagulation are associated with the occurrence of intraplaque hemorrhage, while statins are associated less with intraplaque hemorrhage: a large histopathological study in carotid and femoral plaques. Atherosclerosis.

